# 5-Fluorouracil resistant CRC cells derived exosomes promote cancer-associated fibroblasts secreting more CXCL12

**DOI:** 10.7150/jca.95248

**Published:** 2024-04-29

**Authors:** Tongxin Zhang, Zilong He, Xiaowei Qi, Yu Zhang, Yankui Liu, Linfang Jin, Teng Wang

**Affiliations:** 1 Department of Oncology, Affiliated Hospital of Jiangnan University, Wuxi 214122, Jiangsu, China.; 2 Wuxi Medical College, Jiangnan University, Wuxi 214122, Jiangsu, China.; 3 Department of Pathology, Affiliated Hospital of Jiangnan University, Wuxi 214122, Jiangsu, China.; 4 Department of Pathology, Wuxi No. 9 People's Hospital, Wuxi 214062, Jiangsu, China.

**Keywords:** Colorectal cancer, Cancer-associated fibroblast, Chemoresistance, Transient receptor potential canonical 5, CXCL12

## Abstract

**Background:** Chemoresistance is a key reason for treatment failure in colorectal cancer (CRC) patients. The tumor microenvironment of chemoresistant CRC is distinctly immunosuppressive, although the underlying mechanisms are unclear.

**Methods:** The CRC data sets GSE69657 and GSE62080 were downloaded from the GEO database, and the correlation between TRPC5 and FAP expression was analyzed by Pearson method. The in-situ expression of transient receptor potential channel 5 (TRPC5) and fibroblast activation protein (FAP) in the CRC tissues was examined by immunohistochemistry. TRPC5 expression levels in the HCT8 and HCT116 cell lines and the corresponding 5-fluorouracil (5-FU)-resistant cell lines (HCT8R and HCT116R) were analyzed by western blotting and RT-PCR. Exosomes were isolated from the HCT8R and HCT116R cells and incubated with colorectal normal fibroblasts (NFs), and cancer-associated fibroblasts (CAFs)markers were detected. NFs were also incubated with exosomes isolated from TRPC5-knockdown HCT8R cells, and the changes in intracellular Ca^2+^ levels and C-X-C motif chemokine ligand 12 (CXCL12) secretion were analyzed.

**Results:** TRPC5 and FAP expression showed positive correlation in the datasets. Immunostaining of CRC tissue specimens further revealed that high TRPC5 and FAP expressions were significantly associated with worse tumor regression. Furthermore, chemoresistant CRC cells expressed higher levels of TRPC5 compared to the chemosensitive cells, and knocking down TRPC5 reversed chemoresistance. Exosomes derived from CRC cells induced the transformation of NFs to CAFs. However, TRPC5-exosomes derived from chemoresistant CRC cells can promote CAFs to secrete more CXCL12.

**Conclusion:** Chemoresistant CRC cells can induce CAFs activation and promote CXCL12 secretion through exosomal TRPC5.

## Introduction

CRC is one of the most commonly diagnosed malignancies worldwide 1, and ranks fourth and fifth in terms of incidence and mortality rates respectively in China 2. Drug resistance is a key reason for treatment failure in CRC, and elucidating the mechanisms underlying chemoresistance can improve the therapeutic outcomes.

The tumor immune microenvironment (TIME) can reprogram tumor occurrence, growth, invasion, metastasis and response to treatment 3. Studies show that compared to chemosensitive colorectal tumors, the microenvironment of chemoresistant CRC experiences stronger immunosuppression after chemotherapy, and removing the immunosuppressive cues can reverse chemoresistance 4, 5. The TIME comprises of NFs, multiple immune cell types, and other cells. NFs can be activated by different factors within the tumor microenvironment to transform into the CAFs 6, 7. Several studies have identified distinct CAFs subpopulations in the TIME, which may exert completely opposite effects 8-12. For instance, a specific CAFs subpopulation has been identified that suppresses anti-tumor immunity by secreting CXCL12 and other chemokines 13, and desensitizes the tumor cells to 5-FU 14, 15.

Exosomes are membrane-bound vesicles with diameters ranging from 40-160 nm that are secreted by multiple cell types. The exosomes transport mRNAs, proteins and other bioactive molecules from the mother cells to the recipient cells, wherein they regulate biological functions 16. Studies have confirmed that NFs can be activated by exogenous exosomes and undergo functional changes to transform into CAFs 17. In a previous study, we found that TRPC5 induces chemoresistance in CRC cells 18. Furthermore, chemoresistant breast cancer cells transport TRPC5 to the chemosensitive cells through exosomes 19, 20. The objectives of the present study were to explore the differences between the CAFs of chemosensitive and chemoresistant CRC tissues, and to assess the effects of CRC-derived exosomes on NFs. We found that colorectal NFs formed distinct CAFs subpopulations following uptake of different exosomes from the chemoresistant and chemosensitive CRC cells. Our findings provide new insights into the mechanisms of chemoresistance and immune evasion in CRC.

## Materials and methods

### Cell culture

The HCT8 (CTCC-400-0163) and HCT116 (CTCC-001-0002) cell lines, the corresponding 5-FU-resistant CRC cell lines [HCT8R (CTCC-0206-NY) and HCT116R (CTCC-0213-NY)], and the normal colorectal fibroblast line CCD-Co18 (CTCC-241-HUM) were purchased from MeisenCTCC (Zhejiang, China). HCT8 and HCT8R cells were cultured in RPMI-1640 medium (CTCC-002-123, MeisenCTCC), and CCD-Co18 cells were cultured in DMEM (CTCC-002-084, MeisenCTCC). In addition, the HCT8R and HCT116R cells were cultured in DMEM supplemented with 0.5µg/ml and 0.8µg/ml 5-FU respectively. The culture media for all cell lines were supplemented with 10% fetal bovine serum (FBS; CTCC-002-020, MeisenCTCC), and the cells were maintained at 37°C under 5% CO_2_.

### Transfection

The CRC cells were seeded in a 6-well plate at the density of 30 x 10^5^ cells/well. Once the cells were ~50% confluent, the culture medium was discarded and replaced with serum-reduced transfection medium (abs9461, Absin). The cells were transfected with siRNA construct targeting TRPC5, and gene knockdown at the mRNA and protein level were verified 24 h and 48 h post-transfection respectively. The siRNA sequence of TRPC5 is shown in Table 1.

### CCK-8 assay

The CRC cells were seeded in a 96-well plate at the density of 10^5^ cells/well, and cultured in the presence of 0µM, 125µM, 250µM, 375µM, 500µM, 625µM, 750µM, 875µM, 1000µM, and 1250µM 5-FU. After culturing for 48 h, 10µl CCK-8 reagent was added to each well, and the cells were further incubated for 30 min. The absorbance was measured at 450 nm to determine cell viability.

### Western blotting

Western blotting was performed as previously described 18. Briefly, equal amounts of total protein per sample were separated on a polyacrylamide gel containing 0.1% SDS and then transferred to PVDF membrane. The membranes were incubated overnight with primary antibodies of TRPC5 (1:1000, ACC-020, Alomone Labs), β-actin (1:1000, #4967, Cell Signaling Technology), TSG101 (1:1000, ab125011, Abcam), CD81 (1:1000, ab109201, Abcam), fibroblast activation protein (FAP) (1:1000, ab53066, Abcam), a-SMA (1:1000, ab32575, Abcam), FSP-1 (1:1000, ab124805, Abcam) and Vimentin (1:1000, ab193555, Abcam) at 4°C, followed by horseradish peroxidase (HRP)-conjugated goat anti-rabbit IgG (1:500, ab205718, Abcam) for 1 h at room temperature. The protein bands were developed with an enhanced chemiluminescence kit (E411-04, Vazyme), and observed using ChemiDocXRS+ gel imager (Bio-Rad).

### Real time PCR

Total RNA was isolated from the cells using FastPure® Cell/Tissue Total RNA Isolation Kit V2 (RC112, Vazyme), and 1μg RNA per sample was reverse transcribed into cDNA using HiScript III All-in-one RT SuperMix Perfect for qPCR (R333, Vazyme). RT-PCR was performed on a LightCycler® 480II real-time PCR system (Roche) using ChamQ Universal SYBR qPCR Master Mix (Q711, Vazyme). Gene expression was calculated using the 2^-ΔΔCt^ method, and β-actin was the internal control. The primers are listed in Table 2.

### Exosome extraction

Once the cultured cells were ~50% confluent, the medium was discarded and fresh medium containing exosome-free serum (EXO-FBS-50A-1, System Biosciences) was added. The culture supernatant was collected 48 h later and passed through a 0.22µm filter (SLGPR33RB, MECK). The filtrate was centrifuged at 3000g for 30 min, and the supernatant was mixed with ExoQuick-TC (EXOTC50A-1, System Biosciences) and left undisturbed overnight at 4°C. The solution was centrifuged at 1500 g for 15 min, and the exosome precipitate was collected.

### ELISA

CXCL12A levels in the culture supernatants were measured using the Human SDF-1a (CXCL12A) ELISA Kit (EHCXCL12A, Thermo Fisher). As per the instructions, the test sample was added to the reaction well, followed by the biotin conjugate and streptavidin HRP. The absorbance was measured at 450 nm, and the concentration of CXCL12 was determined by plotting a standard curve.

### Fluo-4 staining

CCD-Co18 cells were seeded in a 6-well plate and cultured till the confluency was ~50%. The medium was replaced with serum-free medium containing the isolated exosomes, and the cells were cultured for 48 h. The cells were then stained with 4µM Fluo-4 AM (40704ES50, Yeasen) and observed under a fluorescence microscope at the excitation wavelength 475 nm and emission wavelength 560 nm.

### Immunofluorescence

The cells were seeded in a 12-well plate on cover slips at the density of 10^5^ cells/well, and cultured for 24 h. After fixing with 4% paraformaldehyde, the cells were incubated with anti-a-SMA (1:500, ab32575, Abcam), anti-FAP (1:500, ab53066, Abcam), and anti-FSP-1 (1:500, ab32575, Abcam), and anti-Vimentin (1:500, ab193555, Abcam) antibodies, followed by Alexa Fluor® 647-conjugated donkey anti-rabbit IgG H&L (1:500, ab150075, Abcam). The immunostained cells were visualized under a fluorescence microscope.

### Bioinformation analysis

The CRC data sets GSE69657 and GSE62080 were downloaded from Gene Expression Omnibus (GEO) database (https://www. ncbi.nlm.nih.gov/geo), and the correlation between TRPC5 and FAP was analyzed by Pearson method using the R package "ggpubr".

### Patients and follow-up

A total of 41 CRC patients who received preoperative neoadjuvant chemotherapy at Jiangnan University Affiliated Hospital from May 2019 to July 2023 were selected. The inclusion criteria were as follows: (1) complete pathology report of colonoscopy biopsy before chemotherapy and paraffin-embedded tumor tissue biopsy; (2) CT and/or MRI showing local progression and no distant metastasis at initial diagnosis; (3) 2-4 cycles of FU-based preoperative neoadjuvant chemotherapy; (4) radical CRC surgery after chemotherapy; (5) complete postoperative pathology report, including TRG report. The clinical and pathological data of the patients were collected, along with colonoscopy biopsy specimens and postoperative tissue specimens for immunostaining. The AJCC tumor regression grading (TRG) system 21 was used to evaluate chemotherapy response as follows: TRG 0/complete response - absence of any viable cancer cells; TRG 1/ near-complete response - single cells or rare small groups of cancer cells; TRG 2/partial response - residual cancer cells with evident tumor regression, but more prominent than single cells or scattered groups of cancer cells; TRG 3/poor or no response - numerous residual cancer cells with no evident tumor regression. The correlation between the expression levels of TRPC5 and FAP in the CRC tissues, and the correlation of these markers with chemotherapy response were analyzed.

The experiments involving clinical samples were approved by the Ethics Committee of the Affiliated Hospital of Jiangnan University, and the study conformed to the principles outlined in the World Medical Association Declaration of Helsinki. Informed consent was obtained from all participants and the specimens were anonymous.

### Immunohistochemistry

CRC tissue sections were deparaffinized and rehydrated, blocked with 10% bovine serum albumin, and incubated overnight with anti-TRPC5 (1:100, TA371579, Origine) or anti-FAP (1:200, ab53066, Abcam) antibodies at 4°C. The following day, the sections were washed and incubated with anti-mouse or anti-rabbit secondary antibody polymer (abs996, Absin). TRPC5 staining was evaluated as described in our previous studies 20, 22. The staining intensity was scored between 0-3 points as follows: 0 - no staining or weak staining; 1 - moderate staining; 3 - strong staining. The staining range was graded between 0-4 based on the percentage of positively stained cells: 0 - 0%; 1 - 1%-24%; 2 - 25%-49%; 3 - 50%-74%; 4 - 75%-100%. Statistical analysis is performed based on the integral of the product of staining intensity and staining range. Five random fields of view are examined per section, and the final score was the average of five. FAP staining was quantified according to previous literature reports 23, 24. The staining intensity of the stroma was graded as follows: 0 - unstained; 1+ - low intensity; 2+ - medium intensity; 3+ - high intensity. The percentage of FAP-positive fibroblasts was scored as follows: grade 0 - missing or < 1%; grade 1 - 1-10%; grade 2 - 11-50%; grade 3 - 51-75%; grade 4 - 76-100%. The composite IHC score was determined by multiplying the staining intensity score with the percentage of positively stained cells.

### Statistical analysis

Student's t-test and Pearson's Chi-squared test were used to compare data as appropriate. P<0.05 was considered statistically significant.

## Results

### TRPC5 expression correlated positively with the FAP in CRC tissues

**Figure 1:**
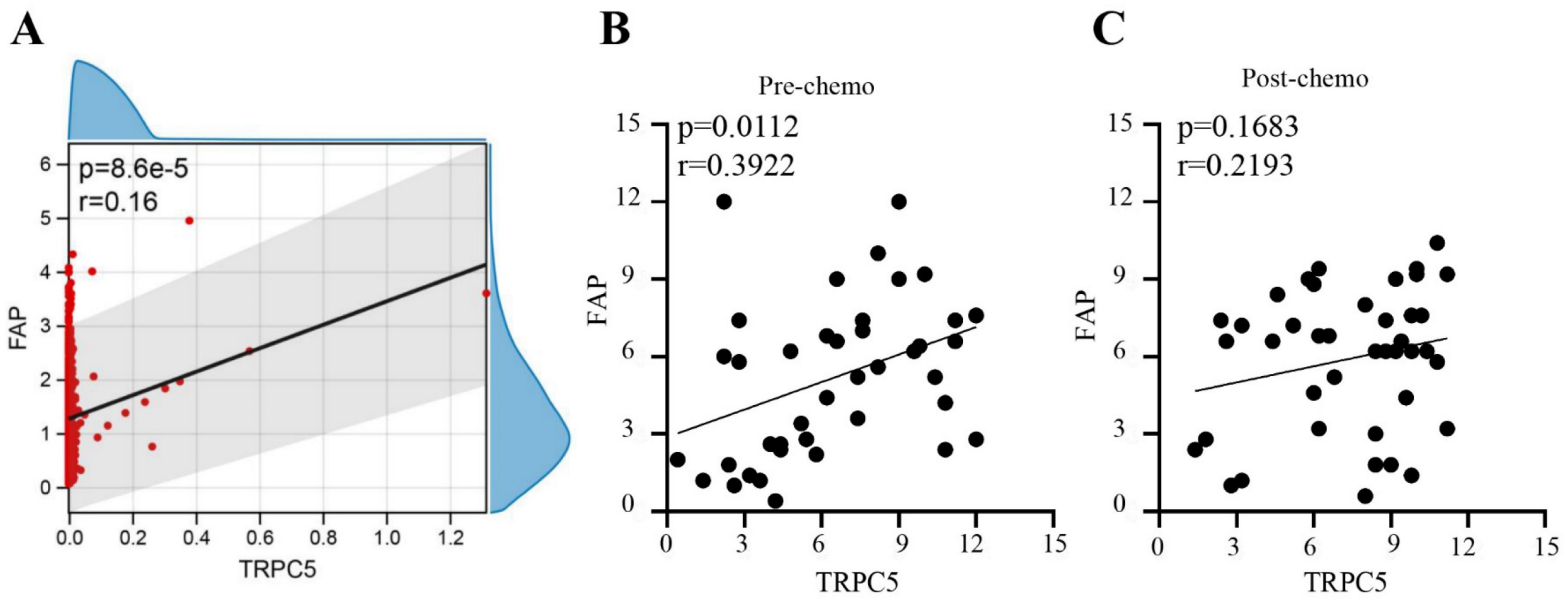
TRPC5 expression correlated positively with the fibroblast activation protein (FAP) in CRC tissues. (A) Bioinformatics analysis shows there is a positive correlation between TRPC5 expression and FAP expression in CRC (p<0.05), (B) Immunohistochemistry detected the expression levels of FAP and TRPC5 in CRC tissue before chemotherapy, and there was a positive correction between the expression levels of TRPC5 and FAP in the colonoscopy biopsies taken before chemotherapy (p<0.05), (C) Immunohistochemistry detected the expression levels of FAP and TRPC5 in CRC tissue after chemotherapy, and no significant correlation was observed in the surgically resected tumor tissues after chemotherapy (p>0.05).

**Figure 2:**
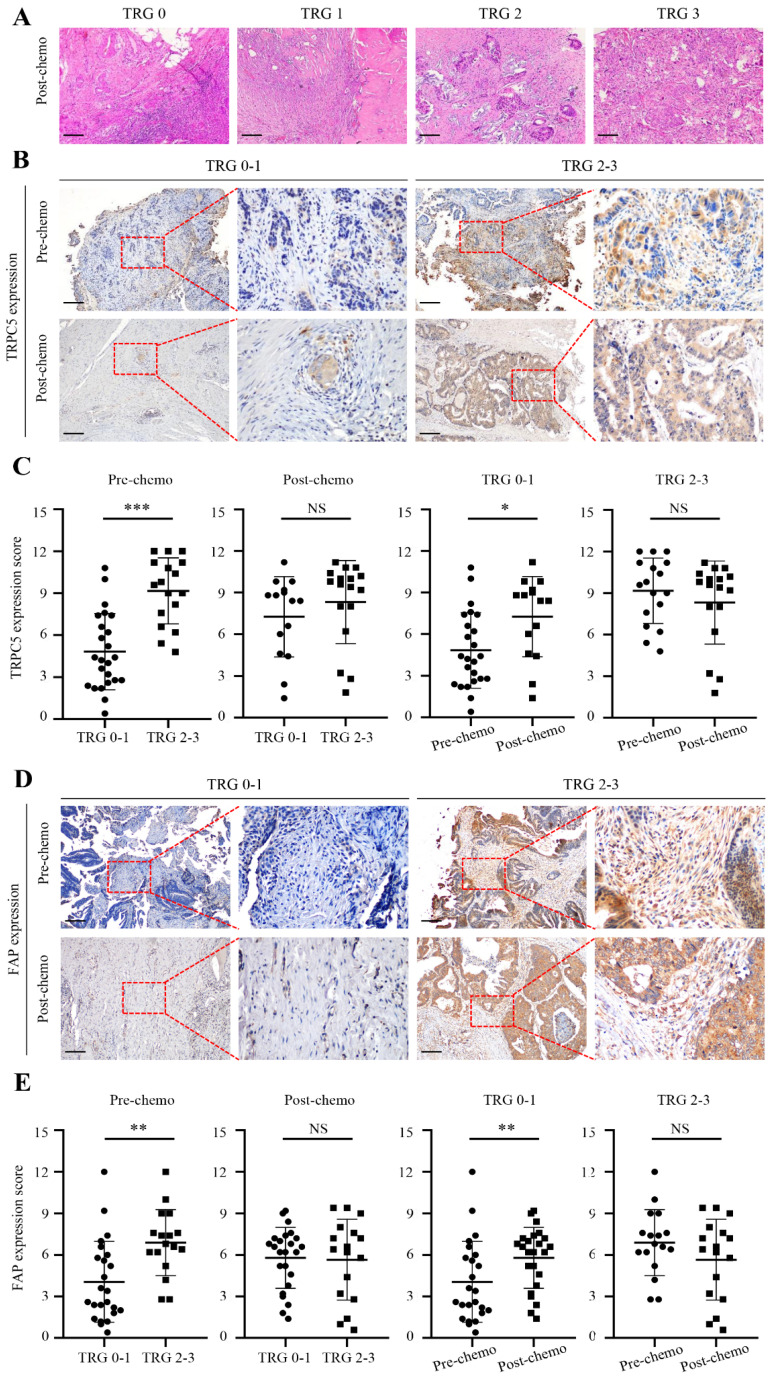
Chemotherapeutic response of CRC cells is related to TRPC5 and FAP expression. (A) Examples of TRG 0-3. (B) Representative images from immunohistochemical staining of TRPC5 expression in CRC tissues according to different TRG grades. Scale bar: 100μm. (C) Student's t-test showed the TRPC5 expression level in CRC tissue before chemotherapy in the TRG 0-1 group was significantly lower than that in the TRG 2-3 group, and increased significantly after chemotherapy, while the change was not observed in TRG 2-3 group. No significant difference in post-chemotherapy CRC tissues between TRG 0-1 and TRG 2-3 group. (D) Representative images from immunohistochemical staining of FAP expression in CRC tissues according to different TRG grades. Scale bar: 100μm. (E) Student's t-test showed the expression level of FAP in CRC tissue before chemotherapy in the TRG 0-1 group was significantly lower than that in the TRG 2-3 group, and increased significantly after chemotherapy, while the change was not observed in TRG 2-3 group. No significant difference in post-chemotherapy CRC tissues between TRG 0-1 and TRG 2-3 group. *p<0.05, **p<0.01, ***p<0.001.

In our previous study, we found that TRPC5 was highly expressed in chemoresistant CRC. Based on the CRC data sets GSE69657 and GSE62080, there is a positive correlation between TRPC5 expression and FAP expression in CRC (Figure 1A). We also verified the expression levels of TRPC5 and FAP in the colonoscopy biopsies and surgically resected CRC tissues respectively collected before chemotherapy and after neoadjuvant chemotherapy from 41 patients with locally advanced CRC (clinical and pathological data are shown in Table 3). Immunostaining of the CRC tissues indicated differences in the expression levels of TRPC5 (Figure 2B) and FAP (Figure 2D). As shown in Figure 1B, there was a positive correction between the expression levels of TRPC5 and FAP in the colonoscopy biopsies taken before chemotherapy (Figure 1B). However, no significant correlation was observed in the surgically resected tumor tissues after chemotherapy (Figure 1C).

### Chemotherapeutic response of CRC cells is related to TRPC5 and FAP expression

Based on postoperative pathological findings, 24 CRC patients had TRG 0-1 and were classified as responders, and 17 patients with TRG 2-3 were classified as non-responders (Figure 2A). Interestingly, the TRG correlated significantly with TRPC5 expression in the CRC tissues before chemotherapy. As shown in Figure 2C, the pre-chemotherapy expression of TRPC5 was significantly lower in the TRG 0-1 group compared to that in the TRG 2-3 group, and increased substantially in the TRG 0-1 group post-chemotherapy, while no changes were observed in the TRG 2-3 group. Furthermore, there was no significant difference in TRPC5 expression between the two groups after chemotherapy (Figure 2C). Likewise, FAP was significantly upregulated in the TRG 0-1 group after chemotherapy, and was consistently high in the TRG 2-3 group (Figure 2E).

### The high expression of TRPC5 in CRC cells is associated with drug resistance

**Figure 3:**
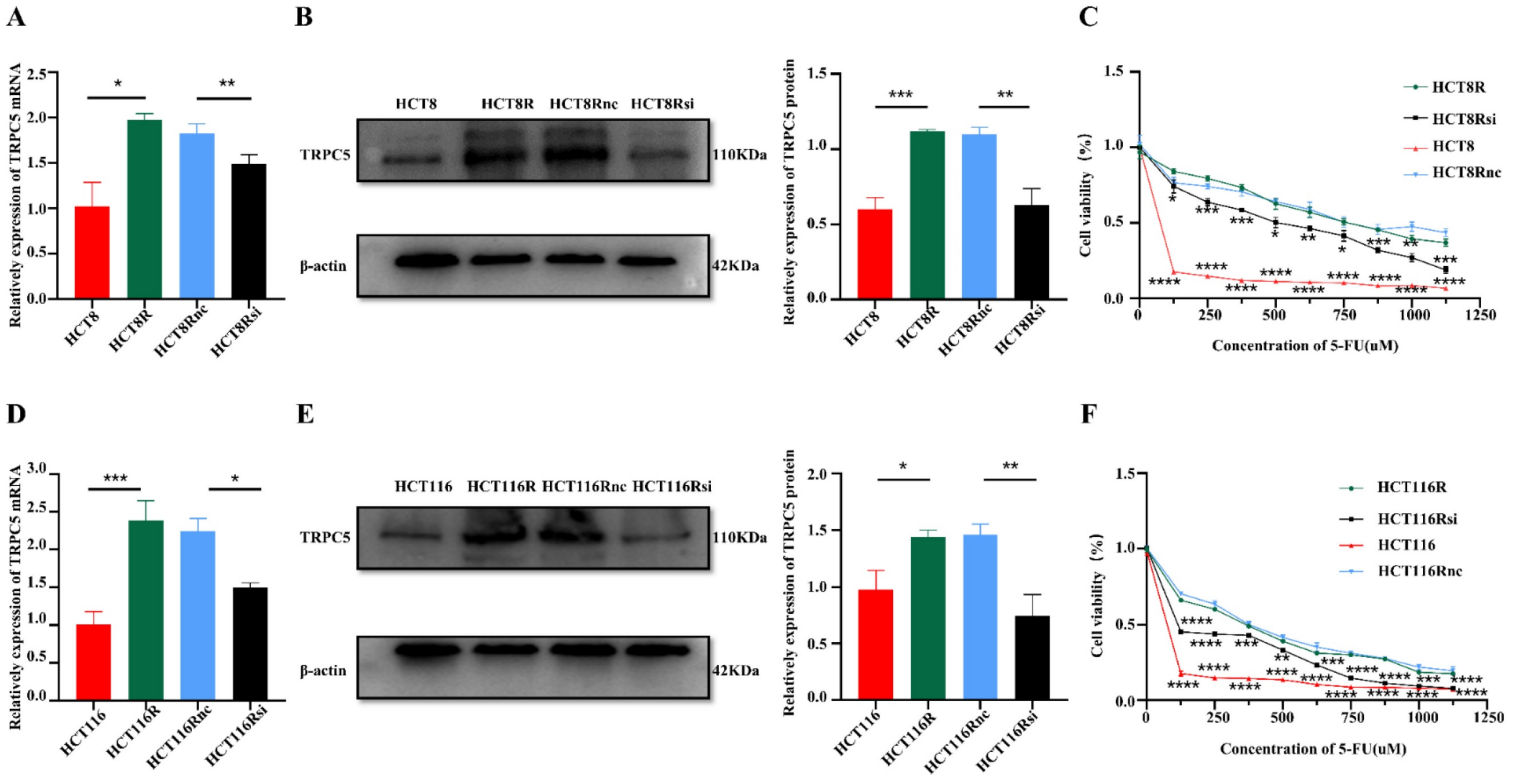
The high expression of TRPC5 in CRC cells is associated with drug resistance. Real time PCR (A, D) showed that the mRNA expression level of TRPC5 in chemoresistant CRC cells is higher than that in chemosensitive CRC cells, and can be significantly reduced by siRNA targeting TRPC5 (N=3). Western blot (B, E) showed TRPC5 protein expression level in chemoresistant CRC cells is higher than that in chemosensitive CRC cells, and can be significantly reduced by siRNA targeting TRPC5(N=3). CCK-8 (C, F) experiment showed that TRPC5 knockdown reduced the chemoresistance index of chemoresistant cells (N=6), and the cell viability of TRPC5 knockdown resistant cells was compared with that of chemotherapy-sensitive cells and chemotherapy-resistant cells. *p<0.05, **p<0.01, ***p<0.001, ****p<0.0001.

One possible explanation of the above findings is that the pressure of chemotherapy increases TRPC5 expression through an unknown mechanism. TRPC5 is a member of the C subgroup of the TRP family and mediates calcium ion influx. We had previously shown that high expression of TRPC5 can induce chemoresistance in CRC cells 18. To further confirm this result, we knocked down the TRPC5 gene in the 5-FU resistant HCT8R and HCT116R cell lines (Figure 3A-F). As shown in Figure 3C and Figure 3F, TRPC5 silencing sensitized the chemoresistant cells to 5-FU and significantly decreased their viability, indicating that TRPC5 is critical for the chemoresistance of CRC cells. Therefore, the post-chemotherapy upregulation in TRPC5 in the CRC cells can also be explained by the survival of TRPC5-overexpressing clones that are resistant to cytotoxic chemotherapy.

### CRC cells can initiate transformation of NFs to CAFs by secreting exosomes

Exosomes reflect the specific molecular signatures of their parent cells 25 and mediate intercellular communication 16. Studies show that NFs can undergo transformation into CAFs following uptake of exosomes 26. Furthermore, different stimulating factors can give rise to activated CAFs subpopulations with distinct functions, such as induction of angiogenesis, promoting inflammation, altering tumor metabolism, and establishing an immunosuppressive tumor microenvironment 27.

**Figure 4:**
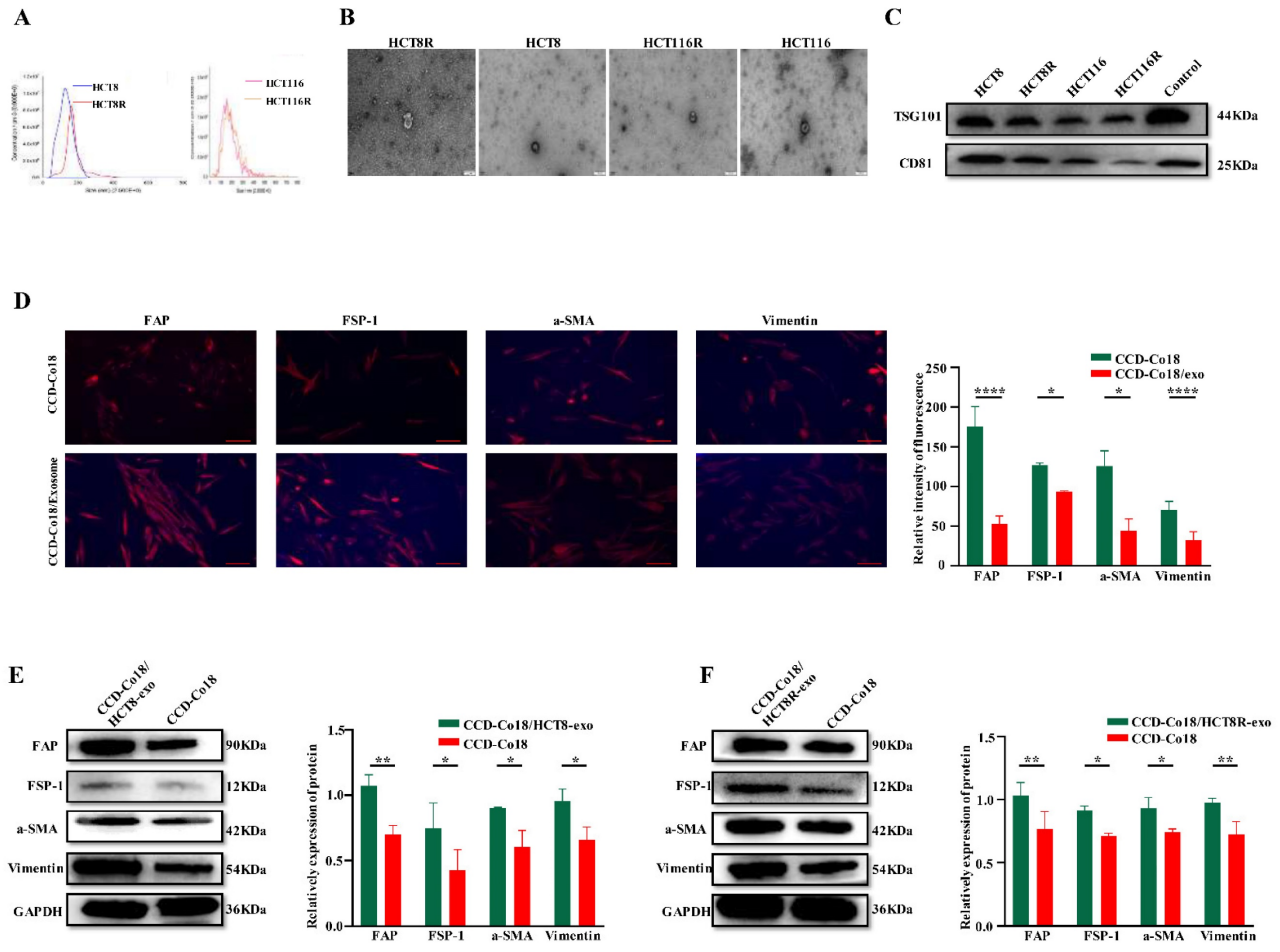
CRC cells can initiate transformation of NFs to CAFs by secreting exosomes. The extracted exosomes were identified by NTA (A) and TEM (B) respectively, and the exosome markers TSG101 and CD81 were detected by Western blot (C). Scale bar: 200nm. (D) Immunofluorescence experiment was performed on normal intestinal fibroblasts and normal intestinal fibroblast markers FAP, FSP-1, a-SMA, and Vimentin after incubation with exosomes for 48 hours, and it was found that they were related to CRCs. The fluorescence signals of these four indicators were stronger in NF co-incubated with exosomes (N=3). Scale bar: 100μm. Western bolt (E-F) showed expression of CAFs markers FAP, FSP-1, a-SMA, and Vimentin increased after incubation with CRC exosomes (N=3). *p<0.05, **p<0.01, ****p<0.0001.

Therefore, we examined whether exosomes derived from chemosensitive and chemoresistant CRC cells can activate NFs into distinct CAFs populations. Exosomes were isolated from the HCT8 and HCT8R cells and characterized by NTA (Figure 4A), TEM examination (Figure 4B), and western blotting (Figure 4C). The respective exosomes were then incubated with the CCD-Co18 cells, and the presence of CAFs was evaluated based on the expression of a-SMA, FSP-1, FAP and Vimentin 7. As shown in Figures 4D-F, exosomes derived from the HCT8R cells (HCT8R-exo) significantly increased the expression of CAFs markers compared to the HCT8 exosomes (HCT8-exo), indicating that exosomes from chemoresistant and chemosensitive tumor cells induce different types of CAFs. Overall, these findings suggested that chemoresistant CRC cells can promote the transformation of NFs to CAFs by secreting exosomes.

### Exosomes secreted from chemoresistant CRC cells deliver TRPC5 to the activated CAFs and promote secretion of CXCL12

**Figure 5:**
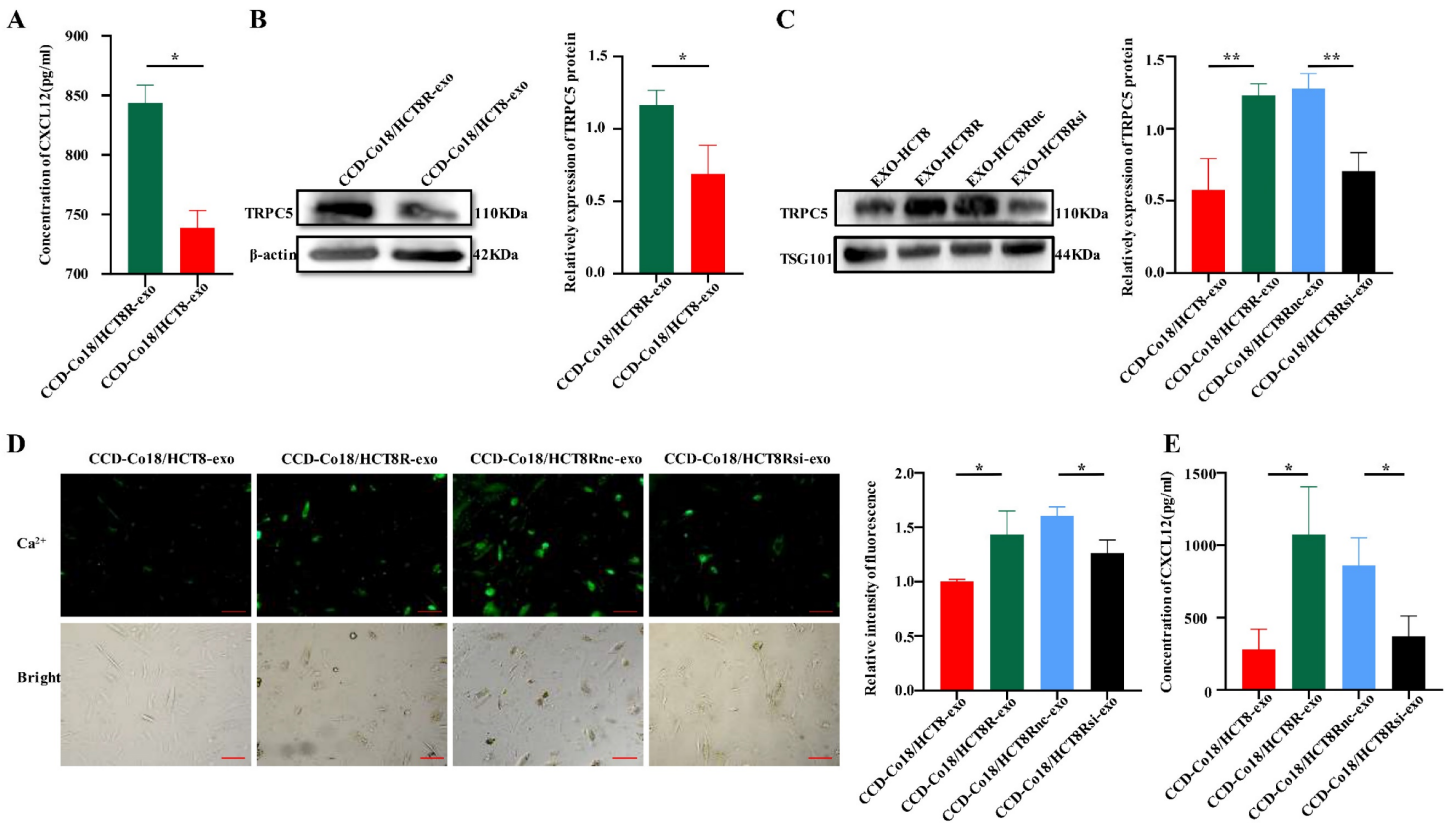
Exosomes secreted from chemoresistant CRC cells deliver TRPC5 to the activated CAFs and promote secretion of CXCL12. (A) ELISA showed the CXCL12 in the culture supernatant of fibroblasts incubated with chemoresistant CRC cell exosomes was higher than that in the culture supernatant of fibroblasts incubated with sensitive CRC exosomes(N=3). (B) Western blot showed higher expression of TRPC5 in CCD-Co18/HCT8R-exo than in CCD-Co18/HCT8-exo (N=3). (C) TRPC5 knockdown in HCT8R resulted in decreased HCT8R exosomes carrying TRPC5 (N=3). (D) Calcium ion imaging showed that the intracellular calcium level of CCD-Co18/HCT8R-exo is higher than CCD-Co18/HCT8 and CCD-Co18/HCT8Rsi-exo (N=3). Scale bar: 100μm. (E) The level of CXCL12 secreted by CCD-Co18/HCT8Rsi-exo was significantly lower than that of CCD-Co18/HCT8Rnc-exo. Knockdown TRPC5 in HCT8R, decreased the exosomes carrying TRPC5 protein, and further led to a decrease in the CXCL12 secretion (N=3). *p<0.05, **p<0.01.

Garg et al. found that a subpopulation of CAFs in pancreatic tumors inhibited the infiltration of cytotoxic T lymphocytes (CTLs) by secreting the chemokine CXCL12^11^. Therefore, we hypothesized that the CAFs induced by HCT8 and HCT8R cells may differ in their ability to secrete CXCL12. To this end, CCD-Co18 cells were respectively incubated with HCT8-exo and HCT8R-exo, and CXCL12 levels in the culture supernatants were measured by ELISA. As shown in Figure 5A, fibroblasts incubated with HCT8R-exo secreted significantly higher amounts of CXCL12 compared to those incubated with HCT8-exo. Thus, chemoresistant CRC cells can induce CXCL12 production in the fibroblasts by secreting exosomes.

TRPC5 promotes the nuclear translocation of hypoxia-inducible factor-1α (HIF-1α) 28, wherein it transcriptionally activates CXCL12^29^. Given that chemoresistant CRC cells overexpress TRPC5 and can deliver the protein to other cells through exosomes 19, 20, we next determined whether exosomes derived from the chemoresistant CRC cells induce activation of CAFs by delivering TRPC5. As shown in Figure 5B, TRPC5 expression was significantly higher in the CCD-Co18 cells incubated with HCT8R-exo (CCD-Co18/HCT8R-exo) compared to the CCD-Co18/HCT8-exo cells. Likewise, HCT8R-exo expressed significantly higher levels of TRPC5 protein compared to HCT8-exo, which suggested that CRC cells can secrete TRPC5 through exosomes. Knocking down the TRPC5 gene in HCT8R cells (HCT8Rsi) decreased exosomal TRPC5 protein expression as well (Figure 5C). Furthermore, CCD-Co18 cells incubated with HCT8Rsi-exo exhibited reduced calcium influx (Figure 5D) and secreted markedly lower levels of CXCL12 (Figure 5E) compared to the CCD-Co18/HCT8R and CCD-Co18/HCT8Rnc-exo cells respectively. Thus, TRPC5 knockdown in the HCT8R cells decreased the number of TRPC5+ exosomes, leading to a decrease in CXCL12 secretion from the CCD-Co18 cells. In summary, chemoresistant CRCs secrete exosomes with high TRPC5 level, and the exosomes activate CAFs to secrete high levels of CXCL12.

## Discussion

At present, 5-FU-based chemotherapy is the mainstay of treating advanced CRC (stages 2, 3, and 4). However, chemoresistance of CRC cells results in treatment failure in most cases. Several mutations and genetic variations have been linked to the development of chemoresistance in cancer cells 30, although only a few have been confirmed by clinical studies 31, most likely due to the differences in vitro systems and clinical specimens. In the randomized GERCOR study conducted on previously untreated CRC patients 32, FOLFIRI (irinotecan, 5-FU, and leucovorin) followed by FOLFOX (oxaliplatin, 5-FU, and leucovorin), or the reverse sequence, achieved > 50% response rates as first-line therapy, while only 4% and 15% of the patients responded to second-line therapy with the two regimes. One possible explanation is that changes in the TIME induced chemoresistance in the tumor cells, thereby reducing therapeutic efficacy. In fact, studies have linked the TIME to chemotherapeutic response and the survival of tumor cells 33. For example, the chemotactic factor CXCL12 is known to promote the resistance of CRC 33 and gastric cancer 14 cells to 5-FU.

The stromal and immune cells in the tumor microenvironment help cancer cells survive in response to various stimuli and therapeutic agents 34, which eventually impact the treatment outcomes 4, 5, 14, 33. For instance, the CAFs modulate tumor progression by secreting cytokines, chemokines, and growth factors 35. In addition, CAFs also create an immunosuppressive milieu by secreting CXCL12, which inhibits the infiltration of CTLs, recruits and activates T regulatory cells (Tregs), and induces polarization of monocytes into the M2 macrophages as opposed to the M1 macrophages 11, 12, 36. The variations in the TIME of chemoresistant and chemosensitive CRC may explain the different treatment outcomes, although the exact mechanism has not been fully elucidated.

We had previously shown that TRPC5 is highly expressed in CRC tissues and induces chemoresistance 18, 37. Furthermore, TRPC5 also promotes nuclear translocation of HIF-1α by regulating calcium ion influx 28, leading to the upregulation of CXCL12^29^. In this study as well, TRPC5 overexpression in the CRC tissues correlated with increased chemoresistance. Furthermore, the expression level of TRPC5 prior to chemotherapy was significantly related to the therapeutic efficacy, and the CRC cases with high TRPC5 expression had a higher proportion of non-responders. Similar trends were observed with FAP expression, and there were more non-responders among the cases with FAP high expression. In fact, the pre-chemotherapy expression levels of TRPC5 and FAP were positively correlated, and both were upregulated in the responders after chemotherapy.

In our previous studies, we had found that chemoresistant tumor cells deliver functional TRPC5 to chemosensitive tumor cells through exosomes and allow the latter to acquire chemoresistant properties 19, 20. However, the exact role of TRPC5 in the chemoresistant tumor microenvironment was not evaluated. To this end, we analyzed whether CRC cell-derived exosomes are taken up by the NFs, and found that exosomes derived from both chemoresistant and chemosensitive CRC cells induced the transformation of NFs into CAFs. However, the exosomes from chemoresistant CRC cells induced higher expression of functional TRPC5 and CXCL12 in the CAFs compared to the chemosensitive CRC exosomes. This suggested that chemosensitive and chemoresistant CRC cells induce CAFs subpopulations with different immunosuppressive functions. Furthermore, TRPC5 knockdown in chemoresistant CRC cells decreased TRPC5 protein levels in the exosomes, and the co-incubated CAFs not only showed a decrease in TRPC5 expression but also secreted significantly lower levels of CXCL12. Altogether, these findings indicate that TRPC5 induces CXCL12 secretion by the activated CAFs.

This study also has some limitations that ought to be considered. We did not elaborate the mechanism through which TRPC5+ exosomes of chemoresistant CRC cells induce CAFs activation and increase CXCL12 secretion. The potential role of HIF-1α will be the focus of our follow-up study.

## Conclusion

In conclusion, we found a positive correlation between the expression of TRPC5 and FAP, a CAFs marker, in CRC tissues, and their high expression was associated with CRC chemotherapy resistance. By further investigation, we found that CRC exosomes were the key factors leading to CAFs activation, and CAFs activated by CRC-resistant TRPC5 exosomes were able to secrete more CXCL12. Therefore, our study partly elucidated the possibility that TIME of drug-resistant CRC may be more immunosuppressive than that of chemotherapy-sensitive CRC. These findings further clarify the role of TRPC5 in chemoresistance of CRC, and may provide new ideas for reversing immunosuppression and improving the efficacy of chemotherapy in drug-resistant CRC.

## Figures and Tables

**Table 1:** Sequences of Si-TRPC5

Gene	Sense	Antisense
Si-TRPC5-NC	5'-UUCUCCGAACGUGUCACGUTT-3'	5'-ACGUGACACGUUCGGAGAATT-3'
Si-TRPC5-1	5'-GCAAUCAAAUACCACCAGAAATT-3'	5'-UUUCUGGUGGUAUUUGAUUGCTT-3'
Si-TRPC5-2	5'-GCUCCUCAAAUUCUAAACUUUTT-3'	5'-AAAGUUUAGAAUUGAGGAGCTT
Si-TRPC5-3	5'-CCUGCUCAUUGCCAUUGAGAATT-3'	5'-UUCUCAAUGGCAAUGAGCAGGTT-3'

**Table 2:** Real time PCR primers

Gene	Forward (5'-3')	Reverse (5'-3')
GAPDH	TGAGCCCTCATTGCCATTG	GTGTCGCTGTTGAAGTCAGAGGAG
TRPC5	AGTGCCCTGCTCATTGCCATTG	GCCCACCACTTCCTTGCGTATG

**Table 3:** Clinical and tumor characteristics of the 41CRC patients

	All Patients (n=41)	
Characteristics	n	%
Age (years)		
Mean	60.0	
SD	10.8	
≤60	19	46.3
>60	22	53.7
Sex		
Male	24	58.5
Female	17	41.5
Tumor location		
Colon cancer	13	31.7
Rectal caner	28	68.3
T stage		
T0	10	24.4
T1	7	17.1
T2	12	29.3
T3	12	29.3
N stage		
N0	30	73.2
N1	6	14.6
N2	5	12.2
Tumor differentiation		
Well or moderately	21	51.2
Poorly	11	26.8
TRG grade		
0	9	22.0
1	15	36.6
2	12	29.3
3	5	12.2

## Abbreviations

CAFs: cancer associated fibroblasts
NFs: normal fibroblast
TIME: tumor immune microenvironment
TRPC5: transient receptor potential channel 5
CXCL12: C-X-C motif chemokine ligand 12
CRC: colorectal cancer
5-FU: 5-Fluorouracil
HIF-1α: hypoxia-inducible factor-1α
